# Evidence-Based Treatments for Adults with Migraine

**DOI:** 10.1155/2015/629382

**Published:** 2015-12-29

**Authors:** Rubesh Gooriah, Randa Nimeri, Fayyaz Ahmed

**Affiliations:** Department of Neurology, Hull Royal Infirmary, Hull, UK

## Abstract

Migraine, a significantly disabling condition, is treated with acute and preventive medications. However, some individuals are refractory to standard treatments. Although there is a host of alternative management options available, these are not always backed by strong evidence. In fact, most of the drugs used in migraine were initially designed for other purposes. Whilst effective, the benefits from these medications are modest, reflecting the need for newer and migraine-specific therapeutic agents. In recent years, we have witnessed the emergence of novel treatments, of which noninvasive neuromodulation appears to be the most attractive given its ease of use and excellent tolerability profile. This paper reviews the evidence behind the available treatments for migraine.

## 1. Introduction

Migraine has a lifetime prevalence of around 15% of the population, affecting women (18%) more than men (8%) [1]. It has been termed the seventh disabler due to its considerable impact on the quality of life (QOL) of patients [2]. A subset of patients progresses from having episodic migraine (EM) to chronic migraine (CM), the latter affecting 1%-2% of the population [3]. This is a gradual process, initially changing from low-frequency EM to a high-frequency stage and eventually to CM [4]. CM is defined as a headache on ≥15 days per month for ≥3 months, of which ≥8 days meets the criteria for migraine with or without aura or responds to migraine-specific treatment [5]. Migraines have significant psychological, social, and economic impacts [6]. Around 75% of patients experience impaired functioning during an attack and around half of them require help from others [6]. In addition to direct healthcare costs, the disorder results in loss of 20 million working days in England [6]. The economic annual impact of migraines is considerable and has been estimated at €27 billion in European countries [7]. The indirect costs of migraine exceed the direct costs [8] and therefore reducing the burden of this disabling condition should be an obvious healthcare intervention target. The management of migraine relies on adequate and prompt alleviation of the pain and on the reduction or complete abolition of the attacks. Several acute and prophylactic treatments are indicated for the treatment of migraines. However, a small proportion of patients suffer from intractable migraines, whereby their attacks are inadequately controlled despite having tried a range of medications. It is interesting to note that, of the available pharmacological prophylactic treatments, none have been designed specifically for this purpose. Migraine is therefore a major public health problem which is not always effectively managed, indicating the need for additional migraine-specific drugs. This paper will review the evidence supporting the pharmacological options together with less commonly used invasive and noninvasive treatments.

## 2. Pathophysiology of Migraine

Migraine is a complex condition with an incompletely understood pathophysiology. The early “vascular theory” popularised the notion that the migraine aura was due to hypoxemia secondary to vasoconstriction and that the headache was the result of rebound vasodilatation [9]. However, when it was found that reduced blood flow was still present at the onset of headaches, it became evident that the vascular theory could not account for all the features of migraine with aura [10]. The alternative and widely accepted theory suggests that cortical spreading depression (CSD), a wave a neuronal hyperactivity followed by an area of cortical depression, accounts for the aura [11, 12] and that the headache depends on activation of the trigeminovascular pain pathway [13, 14]. This has been studied in animal models leading to an in-depth knowledge of ionic, neurochemical, and cellular mechanisms [15]. Induction of spreading depression has been shown to cause vasodilation in meningeal vessels by a reflex dependent on trigeminal and parasympathetic pathways [16]. An array of messengers, including substance P and calcitonin gene-related peptide (CGRP) [17], activate or sensitize pain-signalling pathways in relation to CSD in animal models [15]. CSD has also been reported to cause changes in brainstem nociceptive neuronal activity even when the trigeminal pathway has been inhibited [18]. In humans, functional imaging has shown changes in cortical function and blood flow and the patterns of spread are suggestive of CSD [15].

In CM, atypical pain processing, central and peripheral sensitization, cortical hyperexcitability, and neurogenic inflammation all have a role to play [19]. Central sensitization refers to a state in which nociceptive neurons in the spinal and medullary dorsal horn exhibit increased excitability, increased synaptic strength, and enlargement of their receptive fields beyond the original site of inflammation or injury [20]. Peripheral sensitization defines a state in which primary afferent nociceptive neurons display enhanced responsiveness to external mechanical or thermal stimuli at the site of inflammation or injury. Peripheral sensitization of the trigeminal nerve, and the blood vessels supplied by them, accounts for the throbbing pain. This stage of migraine is termed first-order neuron sensitization [21]. Second-order neuron sensitization occurs when sensitization spreads to the second-order trigeminovascular neurons in the spinal trigeminal nucleus, causing scalp hypersensitivity or cutaneous allodynia [21]. Third-order sensitization is the result of sensitization spreading to the thalamus, which causes extracephalic hypersensitivity [21]. Allodynia is therefore the clinical manifestation of second- and third-order neuron sensitization and a marker of migraine progression [22]. There is evidence that allodynia symptoms occur more commonly in patients who have an extensive history of CM [22]. Migraines are mostly nonallodynic initially but become allodynic after a few years with repeated attacks due to sensitization of the trigeminovascular pathway, which results in a lower threshold for activation and therefore more frequent migraine attacks. This makes allodynia a marker of chronification [23]. Cortical hyperexcitability is thought to be another major factor participating in transformation of EM to CM [24]. Increased cortical excitability, compared to subjects with EM and migraine-free controls, has been shown in subjects with CM [25, 26]. Aurora et al. [27] demonstrated that magnetic suppression of perceptual accuracy was significantly reduced in 25 patients with CM compared with subjects with EM and migraine-free controls, indicating increased cortical excitability. Positron emission tomography scan studies were also performed in ten of the patients with CM, and an enhanced metabolism was observed in the pons and right temporal cortex compared to global cerebral metabolism.

## 3. Acute Treatment

The acute management of migraine attacks aims at achieving rapid pain relief. Several drugs have been shown to be effective in placebo-controlled trials. In many patients, simple analgesics are usually sufficient for controlling pain during an attack. However, some individuals have a suboptimal response to analgesics and can be offered other medications such as 5-HT1B/1D receptor agonists, which are migraine-specific. Opiates should be avoided as far as possible for the treatment of acute migraines.

### 3.1. Analgesics

Analgesics are the drugs of first choice for migraines of mild or moderate severity. Aspirin [28, 29], paracetamol [30], ibuprofen [31], naproxen [32], diclofenac [33], phenazone [34], and tolfenamic acid [35] have all demonstrated efficacy in placebo-controlled trials. Paracetamol has the advantage of causing less gastric irritation. Ibuprofen is probably the most widely used nonsteroidal anti-inflammatory drug (NSAID) and, while is at least as effective as aspirin, it may also cause less gastric side effects. Diclofenac has a rapid onset of action whilst naproxen, which has a longer half-life, has a slower onset of action. Effervescent acetylsalicylic acid has a faster onset of action (depending on the formulation) than regular tablets and has shown efficacy similar to that of sumatriptan 50 mg. In addition, the combination of aspirin, paracetamol, and caffeine is more effective than either drug taken alone or in combination without caffeine [36–38]. Valdecoxib, a COX-2 inhibitor, has been shown to be effective for acute migraine treatment [39]. These analgesics should be taken as soon as the headache starts. The concomitant use of an antiemetic is recommended to treat nausea [40]; also antiemetics are believed to improve the absorption of analgesics [41–43]. There is no evidence, however, that the combined use of an antiemetic with an analgesic is more effective than the analgesic alone. To avoid medication overuse headache (MOH), patients should use these analgesics as infrequently as possible and on less than 15 days per month.

### 3.2. 5-HT1B/1D Receptor Agonists (Triptans)

The so-called “triptans” are considered to be the gold standard symptomatic treatment. They are considered as a second line when patients have inadequate response to simple analgesics. Seven triptans, all of which have strong evidence of efficacy, are marketed, namely, sumatriptan, zolmitriptan, naratriptan, rizatriptan, almotriptan, eletriptan, and frovatriptan. They are thought to act via three potential mechanisms: cranial vasoconstriction [44], peripheral neuronal inhibition [45], and inhibition of transmission through second-order neurons of the trigeminocervical complex [46]. However, they differ in terms of their pharmacodynamics and pharmacokinetic properties and are therefore expected to exhibit differences regarding their efficacy and tolerability profile. With the exception of naratriptan, the oral triptans provide headache relief within 30 to 60 minutes. Response rates at 2 hours range from 50% to 80%, with 20% to 50% of patients being pain-free [47, 48]. A meta-analysis of 53 trials [49] showed that eletriptan 80 mg and rizatriptan 20 mg have higher 2-hour response rates than sumatriptan 100 mg and that naratriptan 2.5 mg and frovatriptan 2.5 mg have the lowest response rates. Eletriptan has the lowest recurrence rates at 2 hours [49]. If oral intake exacerbates nausea, the use of dissolving tablets (wafers) of rizatriptan 10 mg and zolmitriptan 2.5 mg can be considered. Parenteral formulations can also be useful in patients with nausea or vomiting. Subcutaneous sumatriptan appears to have the best clinical efficacy with a 76% response rate [50]. Sumatriptan and zolmitriptan are also available in the form of nasal sprays. However, the dysgeusia associated with nasal spray can worsen nausea. The tolerability profile of rizatriptan, sumatriptan, zolmitriptan, and eletriptan appears to be largely similar [51]. Triptans are associated with medication overuse headaches, but the withdrawal is shorter in duration and less in severity than other patients using ergots and other analgesics [52]. Contraindications for the use of triptans are untreated arterial hypertension, coronary heart disease, Raynaud's disease, history of ischaemic stroke, pregnancy, lactation, and severe liver or renal failure [40]. Triptans should not be taken on less than 10 days per month to avoid the emergence of MOH.

### 3.3. Ergot Derivatives

Placebo-controlled trials addressing the efficacy of ergot derivatives are scarce, although experience with their use is not lacking, having been available for many years. Data from comparative trials have shown ergot derivatives to be inferior to triptans [53, 54]. Ergotamine tartrate and dihydroergotamine (DHE) are the only two compounds with sufficient evidence of efficacy. Some patients who have an inadequate response to triptans will benefit from DHE, the preferred ergot derivative. It is available as a nasal spray and can also be injected subcutaneously or intramuscularly. The main side effects include nausea, vomiting, paraesthesia, and ergotism. It is contraindicated in cardiovascular and cerebrovascular diseases, Raynaud's disease, arterial hypertension, renal failure, and pregnancy and lactation.

## 4. Preventive Treatment

### 4.1. Oral Pharmacotherapy

The aims of migraine prophylaxis are to reduce migraine frequency, severity, and disability and improve quality of life. The overuse of analgesic medications can lead to the development of chronic migraine. Therefore preventive medications also serve to limit the need for frequent analgesic intake, thereby reducing the risk of migraine chronification.

#### 4.1.1. Topiramate

Topiramate has numerous modes of action that include inhibition of glutamatergic excitatory amino acid transmission, inhibition of voltage-gated calcium channels, enhancement of GABA-evoked currents, fast Na+ channel blockade, and carbonic anhydrase inhibition [55]. It is thought to act on processes that suppress the initiation and propagation of CSD [56]. Its benefits in the prophylaxis of migraine were initially shown in two relatively small randomised placebo-controlled trials [57, 58]. In a multicenter randomised double-blind, placebo-controlled study of 468 patients meeting the IHS criteria for episodic migraine, Brandes et al. [59] found that topiramate at doses of 100 mg/day and 200 mg/day significantly reduced the mean monthly migraine frequency compared to placebo (−2.1, *p* = 0.008 and −2.4, *p* < 0.001, resp.). Silberstein et al. [60] randomised 487 patients to placebo (*n* = 117) or topiramate 50 mg/day (*n* = 125), 100 mg/day (*n* = 128), or 200 mg/day (*n* = 117). The study is likely to have included patients with both episodic and chronic migraines based on the frequency of the attacks. The primary efficacy end-point was a reduction in mean migraine frequency from baseline. Secondary efficacy end points included time to onset of action, proportion of subjects responding to treatment (≥50% reduction in the monthly migraine frequency), mean change in monthly migraine days, and change in number of days per month requiring rescue medication from the end of the prospective baseline phase through the double-blind phase. During the 6-month period, 158 patients withdrew, 83 (52.5%) of which were due to adverse events. Topiramate was associated with a dose-dependent significant decrease in mean monthly migraine frequency from baseline, with a mean reduction of 2.3 days seen with a dose of 200 mg/day, confirming the results from Brandes et al. [59]. The most common AEs observed were paraesthesia, fatigue, anorexia, taste perversion, and nausea. These events seemed to be dose dependent and generally resolved over time or with discontinuation. Topiramate is approved for the prevention of EM by the Food and Drug Administration (FDA) and is recommended by the National Institute for Health and Care Excellence (NICE) guidelines [61], but its use is mainly hampered by its side effect profile.

Topiramate is also effective in the prevention of CM. Its efficacy was initially shown in a small randomised, double-blind, placebo-controlled trial with 28 patients suffering from chronic migraine with overuse of acute medications [62]. Diener et al. [63] included 59 patients with CM (32 patients receiving topiramate and 27 receiving placebo) as defined by the ICHD-2 criteria [64] in a randomised, double-blind, placebo-controlled trial. The primary efficacy variable was the change in the mean number of monthly migraine days from baseline to the last 4 weeks of the double-blind phase. Topiramate intake resulted in a significant decrease in the mean number of monthly migraine days from 15.5 ± 4.6 at baseline by 3.5 ± 6.3 in the last 4 weeks of the double-blind phase, compared with an increase of 0.2 ± 4.7 days from 16.4 ± 4.4 at baseline for patients receiving placebo (*p* = 0.02). A larger study was needed to confirm this. In a randomised, double-blind, placebo-controlled trial involving 306 patients (topiramate, *n* = 153; placebo, *n* = 153) and consisting of 16 weeks of double-blind treatment, topiramate (titrated to a maximum dose of 100 mg/day) resulted in a statistically significant mean reduction of migraine/migrainous headache days (topiramate −6.4 versus placebo −4.7, *p* = 0.010) and migraine headache days relative to baseline (topiramate −5.6 versus placebo −4.1, *p* = 0.032) compared to placebo [65]. Adverse events were reported in 80.2% of the topiramate-treated group compared to 70.2% in the placebo-treated group leading to discontinuations in 18 (10.9%) topiramate subjects and 10 (6.1%) placebo subjects.

#### 4.1.2. Amitriptyline

There are a few old placebo-controlled trials supporting the use of amitriptyline for migraine prophylaxis, but they are small [66, 67]. The evidence that best supports the use of amitriptyline for migraine prophylaxis emanates from a 26-week, multicenter, randomised, double-blind, double-dummy, parallel-group, noninferiority study comparing it to topiramate [68]. The intent-to-treat population included 331 subjects (172 topiramate, 159 amitriptyline). The primary efficacy outcome was the change from prospective baseline in the mean monthly number of migraine episodes. There was no significant difference between the groups in the least squares mean (LSM) change from baseline in the mean monthly number of migraine episodes (−2.6 and −2.7, resp.). However, topiramate conferred a significantly greater improvement in mean functional disability scores during migraine attacks compared with amitriptyline (LSM change: −0.33 versus −0.19; 95% CI, −0.3 to 0.0; *p* = 0.040) and in the function-restrictive role, function-preventive role, and emotional function domains of the MSQ (*p* = 0.012, *p* = 0.014, and *p* = 0.029, resp.). A mean weight loss of 2.4 kg was observed in subjects receiving topiramate, compared with a mean weight gain of 2.4 kg in subjects receiving amitriptyline. Adverse events were reported in 66.7% and 66.3% of those on topiramate and amitriptyline, respectively.

#### 4.1.3. Beta-Blockers

The benefits of beta-blockers in migraine prevention were noted fortuitously in those who were being treated for hypertension. Propranolol and metoprolol have the best evidence in migraine prophylaxis. Several trials show clear and consistent evidence that propranolol is more effective than placebo, although the majority of these have methodological weaknesses. Its efficacy is nonetheless considered to be well established and has been supported by a Cochrane review [69]. When compared to amitriptyline, propranolol has been found to have a similar efficacy [70]. Most of the trials used the conventional formulation of propranolol. Pradalier et al. [71] showed that the long-acting preparation of propranolol was also effective in reducing the frequency of migraine attacks. Studies with propranolol reported efficacy with doses of 80 to 240 mg, with both conventional and slow-release formulations. Metoprolol has been found to exert a prophylactic effect comparable to that of propranolol [72–74]. With regard to other beta-blockers, the evidence is less convincing. Timolol [75] and nadolol [76] have both been found to be equally effective to propranolol. Bisoprolol, a highly selective beta-1 adrenoceptor antagonist, has a similar efficacy to metoprolol [77] while atenolol has been shown to be superior to placebo [78]. Side effects of beta-blockers include fatigue, hypotension, bradycardia, depression, and vivid dreams. They are contraindicated in asthmatics.

#### 4.1.4. Valproic Acid

Valproic acid is thought to block neurogenic inflammation within the meninges through GABA_A_-mediated receptors [79]. Hering and Kuritzky [80] reported the first double-blind placebo-controlled trial of sodium valproate in migraine prophylaxis. Further randomised placebo-controlled trials using valproic acid (sodium valproate or divalproex sodium) provided additional evidence of its efficacy in migraine prophylaxis [81–83]. Valproic acid has been found to be equally effective to propranolol in a single-investigator, randomised, single-blind, placebo-controlled study [84]. Of the 37 patients recruited, 32 completed the study. Migraine frequency was reduced in 19% (6/32) of placebo-treated, 66% (21/32) of divalproex-treated, and 63% (20/32) of propranolol-treated patients [84]. When compared to flunarizine, valproic acid was also found to have similar efficacy [85]. Freitag et al. [83] found no significant differences between treatment groups either in the overall incidence or in the incidence of any specific treatment-emergent adverse event; 8% of subjects treated with extended-release divalproex sodium and 9% of those treated with placebo discontinued for adverse events [83]. A Cochrane review published in 2013 concluded that valproic acid was effective and well-tolerated in adults with episodic migraine [86]. However, given the teratogenic properties of valproate, it is not recommended in female migraineurs of child-bearing age.

#### 4.1.5. Calcium-Channel Antagonists

Of the calcium-channel antagonists, flunarizine has the best evidence, albeit not Class I, to support its use in migraine prophylaxis. A few placebo-controlled trials are available, but these are small [87–89]. Louis [87] initially reported a statistically significant reduction in migraine attacks with flunarizine compared to placebo among 53 migraineurs. In another double-blind placebo-controlled trial involving 101 patients (flunarizine = 50, placebo = 51), the mean reduction in monthly migraine attacks was 2.3 in the flunarizine and 1.7 in the placebo groups (*p* = 0.018) [88]. Moreover, a phase-IV double-blind equivalence trial completed by 666 patients and designed to assess the efficacy and tolerability of two doses of flunarizine (5 mg and 10 mg o.d.) in the prophylaxis of migraine, in comparison with slow-release propranolol (160 mg o.d.), showed that flunarizine at a dose of 10 mg/day was at least as effective as 160 mg propranolol for primary efficacy parameters such as reduction in mean monthly attack frequency and number of responders [90]. An earlier study involving 488 patients comparing flunarizine and propranolol had shown similar results [91]. The most common side effect of flunarizine is weight gain, with one trial reporting an average gain of 0.6 kg [92]. Unfortunately, flunarizine is not marketed or licensed in the UK, but it is available on a named patient basis from a few centres. Cyclandelate has also been studied but with conflicting results and with the better designed studies being negative [93–97]. The evidence for cinnarizine [98–100] and nimodipine [101–103] is weak, emerging mainly from small placebo controlled, comparative, or open-label studies.

#### 4.1.6. Other Medications

Other antiepileptic drugs, such as gabapentin [104], lamotrigine [105], and oxcarbazepine [106], either are ineffective or lack robust evidence for recommendation as a migraine prophylaxis. Of the angiotensin converting enzyme inhibitors and angiotensin receptor antagonists, candesartan and lisinopril have some evidence in migraine prophylaxis. Candesartan was found to be superior to placebo in reducing secondary endpoint variables such as migraine days, headache severity, and level of disability, in a Class II crossover study involving 57 patients [107]. At a dose of 16 mg per day, candesartan was shown to have similar efficacy to propranolol 160 mg/day in a relatively large comparative study [108]. Lisinopril is supported by a relatively small double-blind placebo-controlled trial showing a statistically significant reduction in the number of headache and migraine days and in headache severity [109]. With regard to antidepressants, whilst amitriptyline has good evidence to support its use, fluoxetine has revealed conflicting results. It was shown to be effective in a small placebo-controlled trial [110], but these results were not replicated in a larger trial [111]. Venlafaxine has been shown to be effective in a randomised, double-blind, placebo-controlled study involving 60 patients with migraine. Extended-release venlafaxine at a dose of 150 mg/day was more effective than 75 mg/day or placebo in reducing the number of headache attacks [112]. A Class II trial assessed the efficacy of venlafaxine versus amitriptyline. Both were effective in reducing the frequency of attacks (venlafaxine: baseline = 4.15 (SD ± 2.24) versus 12 weeks = 1.77 (SD ± 1.39; *p* < 0.001); amitriptyline: baseline = 3.27 (SD ± 1.61) versus 12 weeks 1.54 (SD ± 1.54; *p* < 0.001)) [113]. The serotonin blocker pizotifen (pizotyline) has been evaluated in several placebo-controlled and comparison trials, showing consistent efficacy for migraine prevention [114–118]. However, in the majority of these trials, it was poorly tolerated causing weight gain and sedation and was associated with a high incidence of withdrawals due to adverse events. These have limited its use. Pizotifen is not available in the United States. A technical review of clonidine including 16 trials found that it lacked conclusive evidence of efficacy [119]. Three of the eleven placebo-controlled trials showed a statistically significant improvement over placebo, but the magnitude of this was small [119]. When clonidine was compared to propranolol [120, 121], these trials have yielded mixed results. Herbal remedies, vitamins, and other supplements also have some role in migraine prophylaxis. The root extract of butterbur (*Petasites hybridus*) has been shown to be safe [122] and effective in placebo-controlled trials [123, 124]. A Cochrane systematic review and meta-analysis of studies investigating the effectiveness of feverfew was negative, therefore restricting its use [125]. The evidence supporting the use of riboflavin is scarce, with one placebo-controlled trial showing that a daily dose of riboflavin 400 mg was superior to placebo [126]. Magnesium has been evaluated in 2 placebo-controlled trials for migraine prevention [127, 128] and one assessing its efficacy in menstrual migraine [129]. The first two trials showed benefit over placebo, while the third one failed to show any significant difference. It is worth noting that these three studies measured different endpoints.

### 4.2. Needle Interventions/Injections

#### 4.2.1. Onabotulinumtoxin A

Onabotulinumtoxin A is approved for the treatment of numerous disorders [130]. Its serendipitous benefits as a prophylactic treatment for migraine were observed when it was used as a cosmetic treatment for wrinkles [131]. Freitag et al. [132] carried out a randomised, double-blind, placebo-controlled trial studying onabotulinumtoxin A for CM. Sixty patients were randomised and 100 U of onabotulinumtoxin A was administered in a fixed dose and site paradigm. It was shown to be significantly superior to placebo for the primary end point of reduction in migraine headache episodes. Larger and more robust trials were still needed to confirm these findings, and they came in the form of the Phase III REsearch Evaluating Migraine Prophylaxis Therapy (PREEMPT) trials [133, 134]. These were multicenter, double-blind, placebo-controlled studies conducted to evaluate the efficacy and safety of onaBoNTA for the prophylaxis of headaches in patients with CM. PREEMPT 1 failed to achieve its primary efficacy end-point which was the mean change from baseline in frequency of headache episodes for the 28-day period ending with week 24. With regard to secondary efficacy end points, there was a significant between-group difference in the mean decrease from baseline in the frequency of headache days observed at all time points (−7.8 onaBoNTA versus −6.4 placebo, *p* = 0.006). PREEMPT 2 was methodologically similar to PREEMPT 1, except that the primary efficacy end point of PREEMPT 2 was switched to a mean change from baseline in the frequency of headache days instead of headache episodes. Both PREEMPT studies showed a similar mean change from baseline in frequency of headache episodes in the onaBoNTA group (−5.2 and −5.3, resp.). A pooled analysis (*n* = 1,384) showed a statistically significant mean reduction from baseline in the frequency of headache days favouring onaBoNTA over placebo at week 24 (−8.4 versus −6.6; *p* = 0.01) and all other time points [135]. More importantly, relevant improvements in functioning and health-related quality of life were observed with onaBoNTA compared with placebo in both trials. These improvements were even more manifest in the pooled analyses. The PREEMPT trials have been criticised for the high placebo effect observed, for enrolling a majority of patients with concomitant medication overuse, and for the fact that only 40% of its subjects had previously received a migraine prophylactic [136]. They have nevertheless established the efficacy, safety, and tolerability of onabotulinumtoxin A for CM.

#### 4.2.2. Greater Occipital Nerve Block (GONB)

Peripheral nerve blocks have been practised over the past few years for different forms of headaches. The greater occipital nerve has sensory fibres originating mainly form the C2 segment of the spinal cord [137]. The rationale for using GONB relies on the functional and anatomical continuum between nociceptive trigeminal and upper cervical afferents. Several techniques to perform occipital nerve blocks exist, all of which seem to be effective [138]. However, controlled trials assessing its therapeutic benefits in migraines are lacking, most of them being small and uncontrolled [139, 140]. A study of 97 patients with migraine and 87 with posttraumatic headache who had GONB with a combination of lidocaine and methylprednisolone showed a significant improvement in 54% of migraineurs for up to 6 months [140]. The presence of occipital tenderness increases the likelihood of a positive response. Over the past few years, there has been renewed interest in GONB. However, despite the fact that it is generally safe, potential side effects, such as dizziness, light-headedness and nausea, and rarely cardiac arrhythmias and hypersensitivity reactions, relimit its use [138]. Also, the invasive nature of the procedure makes it less acceptable to patients as a first-line treatment. We nevertheless use GONB effectively in a subset of patients who are refractory to conventional pharmacotherapy.

#### 4.2.3. Acupuncture

Acupuncture, which refers to the application of needles to specific body parts, is widely used in healthcare systems of countries in the East and is well received by the general public. However, despite being introduced as far back as the 17th century in Europe, it is still met with scepticism about its efficacy. Acupuncture has been found to be effective in an array of medical conditions. Several trials have investigated the efficacy of acupuncture for migraine prevention [141–143], and, in recent years, larger and more robust trials have been undertaken [144–147]. The results have been mixed especially when true acupuncture has been compared to sham acupuncture. When acupuncture has been compared with metoprolol, Streng et al. [148] found that the number of migraine days decreased by an average of 2.5 days in the acupuncture group versus 2.2 days in the metoprolol group and that the proportion of responders (reduction of migraine attacks by > or = 50%) was 61% for acupuncture and 49% for metoprolol, although these did not reach statistical significance. This small difference could be explained by the larger placebo effects that accompany physical interventions [149]. Linde et al. [150] felt that sham acupuncture might be associated with larger effects than pharmacological and other “physical placebos” after reanalysing data published in a Cochrane review. Interestingly, Diener et al. [146] randomised 960 patients to verum acupuncture (*n* = 313), sham acupuncture (*n* = 339), or standard therapy (*n* = 308) and found mean reduction of 2.3 days (95% CI 1.9–2.7) in the verum acupuncture group, 1.5 days (1.1–2.0) in the sham acupuncture group, and 2.1 days (1.5–2.7) in the standard therapy group at 26 weeks, but this was not statistically significant across treatment groups. The proportion of responders, defined as patients with a reduction of migraine days by at least 50%, was 47% in the verum group, 39% in the sham acupuncture group, and 40% in the standard group (*p* = 0.133). This again supports the possibility that sham acupuncture achieves more than pharmacological placebo and raises the question of whether sham acupuncture differs significantly from verum acupuncture. A systematic review of clinical trials between 2005 and 2006 revealed that most studies failed to show a statistically significant difference in outcomes, and most of these (13/22 = 59%) found that sham acupuncture may be as efficacious as true acupuncture [151]. This review casts doubts on the importance of accurate needle placement of specific body points during acupuncture. This was acknowledged by a Cochrane review which also concluded that acupuncture is at least as effective as, or possibly more effective than, prophylactic drug treatment and has fewer adverse effects [152].

### 4.3. Neuromodulation

Neuromodulation influences pain signals for the purpose of reversible modification of the nociceptive system function by the exogenous application of electrical currents [153].

#### 4.3.1. Transcranial Magnetic Stimulation

Transcranial magnetic stimulation (TMS) is thought to disrupt CSD by delivering a fluctuating magnetic field from the scalp by which small electrical currents are induced in the brain [154]. There is robust evidence to suggest that single-pulse TMS (sTMS) is effective as an acute treatment for migraine with aura. In a randomised, sham-controlled involving 42 patients, there was a significantly higher pain relief or pain-free rate in the sTMS treated group (69% versus 48%) [155]. A larger randomised, sham-controlled trial involving 164 patients assigned to either sTMS or sham stimulation in a 1 : 1 revealed significantly higher pain-free response rates after 2 h with sTMS (32/82 (39%)) than with sham stimulation (18/82 (22%)) (*p* = 0.0179) [156].

Given that sTMS is effective at treating acute migraine, one can hypothesize that repetitive TMS (rTMS) is beneficial in migraine prevention. Indeed, there is encouraging data to support rTMS for migraine prevention. After the efficacy of high-frequency rTMS was initially demonstrated in a small sham-controlled pilot study [157], Teepker et al. [158] showed a reduction of headache frequency in a study of low-frequency rTMS in 27 patients, but the difference was not significant compared to the sham-treated group. In a randomised, double-blind, placebo-controlled trial, 50 adult migraine sufferers having more than 4 attacks in a month were allocated to either high-frequency rTMS or sham treatment [159]. At 1 month, the rTMS-treated group showed a reduction in headache frequency in 78.7% compared to 33.3% in those receiving sham. Data acquired through years of use of TMS suggest that it is safe. Seizure is rare in patients who use sTMS and is the only adverse event experienced with rTMS to be concerned about, but again the risk is very low [160]. Due to its interaction with some metals, it should be avoided in patients with ferromagnetic implants. The device is chip-activated by a SIM card similar to what is used in mobile phones [161]. The consumer pays for the SIM card while the device remains the property of the manufacturer. The treatment is costly and although it is recommended by NICE, it is yet to be funded in the public sector in the UK. The main disadvantage of TMS is the size of the device, although one can only expect it to shrink with time.

#### 4.3.2. Occipital Nerve Stimulation

Percutaneous occipital nerve stimulation (ONS) is an invasive procedure which was initially investigated in the treatment of chronic cluster headache [162]. Peripheral neurostimulation was found to be effective in an uncontrolled consecutive case series of 25 refractory patients with transformed migraine implanted with C1 through C3 peripheral nerve stimulation [163]. Matharu et al. [164] also reported 8 patients with CM who had a marked improvement with bilateral implanted occipital nerve stimulators. In a prospective, multicenter, blinded, and placebo-controlled study (ONSTIM study), Saper et al. [165] randomised 68 subjects with medically intractable CM to one of three treatment groups—adjustable stimulation (AS), preset stimulation (PS), and medical management (MM)—using a ratio of 2 : 1 : 1, respectively. Subjects who received an implanted device that provided PS rather than AS served as control for the AS group. Included subjects had experienced migraine for an average of 22.0 years prior to the study (range, 1–51 years). A responder was defined as a subject who achieved a 50% or greater reduction in the number of headache days per month or a three-point or greater reduction in average overall pain intensity compared with baseline. Three-month responder rates were 39% for AS, 6% for PS, and 0% for MM. However, lead migration occurred in 12 patients (24%). In another randomised, controlled multicenter study, patients diagnosed with CM were implanted with an occipital nerve stimulator and randomised 2 : 1 to active (*n* = 105) or sham (*n* =  52) stimulation [166]. The study failed to meet its primary endpoint, a difference in the percentage of responders (defined as patients that achieved a ≥50% reduction in mean daily visual analogue scale (VAS) scores) in each group at 12 weeks but showed reductions in pain, headache days, and migraine-related disability. Although the authors also tried to investigate the safety of ONS in CM, the 12-week follow-up is inadequate to answer this. Two other randomised controlled trials are available, one with a small number of patients [167] and the other only published in abstract form [168]. However, the results of all these trials have not been as significant as anticipated and more promising results have been obtained in chronic cluster headaches, although no RCTs have been conducted. For this reason, ONS for CM is only considered on an off-labelled basis. Long-term efficacy and safety of ONS still need to be addressed.

#### 4.3.3. Supraorbital Nerve Stimulation

The first case of intractable cluster headache responding to percutaneous supraorbital nerve stimulation (SONS) was published in 2007 [169]. There are no studies investigating the use of SONS alone in patients with migraine. However, in combination with ONS, it has been used to treat CM [170, 171]. Dual ONS and SONS for CM were studied in fourteen patients with follow-up ranging from 3 to 60 months [170]. Successful stimulation, defined as a 50% or greater reduction in pain severity, was seen in 71%, with a mean reduction in headache-related VAS score of 3.92 ± 2.4. Half of the patients also had resolution of migraine-associated neurological symptoms and returned to normal functional capacity. A high rate of lead migration was observed (42.8%), with other major adverse events being supraorbital lead allodynia (21.4%) and infection (14.2%). The reoperation rate was also high (35.7%). Dual stimulation was superior to the positive response rates (≥50% pain reduction) in the published studies of ONS for CM. Reed et al. [171] described 7 patients with CM, 3 of which met Schulman's criteria for refractory migraines [172], in whom bilateral combined ON-SON stimulators were implanted [171]. All of the patients reported significant improvement in headache frequency and severity and return to a fully active lifestyle. However, larger randomised controlled trials with long-term follow-up are needed to confirm its efficacy and assess its safety.

More promising is the development of a new noninvasive supraorbital transcutaneous stimulator (STS) for migraines. The prevention of migraine using the Cefaly device (PREMICE Study) was a randomised, sham-controlled trial assessing the efficacy and safety of trigeminal neurostimulation with a STS [173]. Sixty-seven patients were randomised to either verum or sham stimulation with the stimulator applied for 20 minutes daily for 3 months. The primary outcome of 50% responder rate was significantly higher (38.1%) in the verum group than in the sham group (12.1%). The Cefaly device was found to be safe and well-tolerated in a patients' satisfaction survey involving 2313 participants, with 53.4% of patients willing to purchase the device [174]. The US Food and Drug Administration (FDA) granted approval for the marketing of the Cefaly device in March 2014. It is small and reasonably priced [175]. This is currently being appraised by NICE although funding through the NHS remains doubtful.

## 5. Conclusion

There is array of options for the acute and prophylactic management of migraine; some are more effective than others. The choice of a prophylactic agent should be tailored to the patient's needs and expectations, bearing in mind that their side effects can differ. We recommend the use of topiramate, amitriptyline, and propranolol as first-line prophylactics. However, in the future, it is likely that the noninvasive options such as TMS and Cefaly will become more widely accessible as prices are lowered. Invasive techniques such as percutaneous ONS or SONS should be reserved for those who have failed to respond to or did not tolerate all conventional treatments due to the risks associated with these interventions and the lack of long-term data on safety and efficacy.
